# Adapting iron dose supplementation in pregnancy for greater effectiveness on mother and child health: protocol of the ECLIPSES randomized clinical trial

**DOI:** 10.1186/1471-2393-14-33

**Published:** 2014-01-18

**Authors:** Victoria Arija, Francesc Fargas, Gemma March, Susana Abajo, Josep Basora, Josefa Canals, Blanca Ribot, Estefania Aparicio, Nuria Serrat, Carmen Hernández-Martínez, Núria Aranda

**Affiliations:** 1 Unitat de Suport a la Recerca Tarragona-Reus, Institut d’Investigació en Atenció Primària Jordi Gol, Tarragona, Spain; 2 Research Group in Nutrition and Mental Health (NUTRISAM), Institut d’Investigació Sanitària Pere Virgili (IISPV), Universitat Rovira i Virgili, C/Sant Llorenç 21, Reus 43201, Spain; 3 Servei d’Atenció Sexual i Reproductiva de Reus-Tarragona, Institut Català de la Salut, Generalitat de Catalunya, Tarragona, Spain; 4 Laboratorio Clínico del Hospital Universitari Joan XXIII, Institut Català de la Salut, Generalitat de Catalunya, Tarragona, Spain; 5 CIBERobn (Center for Biomedical Research in Physiopathology of Obesity and Nutrition), Institute of Health Carlos III, Madrid, Spain

## Abstract

**Background:**

Currently, there is no consensus regarding iron supplementation dose that is most beneficial for maternal and offspring health during gestation. Recommended iron supplementation dose does not preempt anemia in around 20% of the pregnancies, nor the risk of hemoconcentration in 15%. This deficit, or excess, of iron prejudices the mother-child wellbeing. Therefore the aims of the study are to determine the highest level of effectiveness of iron supplementation adapted to hemoglobin (Hb) levels in early pregnancy, which would be optimum for mother-child health.

**Methods/Design:**

*Design*: Randomized Clinical Trial (RCT) triple-blinded

*Setting*: 10 Primary Care Centers from Catalunya (Spain)

*Study subjects*: 878 non-anemic pregnant women at early gestation stage, and their subsequent newborns

*Methods*: The study is structured as a RCT with 2 strata, depending on the Hb levels before week 12 of gestation. Stratum #1: If Hb from 110 to 130 g/L, randomly assigned at week 12 to receive iron supplement of 40 or 80 mg/d. Stratum #2: If Hb >130 g/L, randomly assigned at week 12 to receive iron supplement of 40 or 20 mg/d.

*Measurements*: In the mother: socio-economic data, clinical history, food item frequency, lifestyle and emotional state, and adherence to iron supplement prescription. Biochemical measurements include: Hb, serum ferritin, C reactive protein, cortisol, and alterations in the HFE gene (C282Y, H63D). In children: ultrasound fetal biometry, anthropometric measurements, and temperament development.

Statistical analyses, using the SPSS program for Windows, will include bivariate and multivariate analyses adjusted for variables associated with the relationship under study.

**Discussion:**

Should conclusive outcomes be reached, the study would indicate the optimal iron supplementation dose required to promote maternal and infant health. These results would contribute towards developing guidelines for good clinical practice.

**Trial registration:**

This clinical trial is registered at http://www.clinicaltrialsregister.eu as EudraCT number 2012-005480-28

## Background

### Background and rationale

The prevalence of anemia during pregnancy is high (25%), even in economically developed industrialized countries [1-3]. Also, there is a frequent risk of hemoconcentrations in about 15% of pregnancies [2,4,5].

Ferropenic anemia during pregnancy has been related to an increase in risk of low birth weight of the newborn, premature birth, or less cognitive development of the child [1,5-8].

On the other hand, hemoconcentration, defined in a recent Cochrane review as values of hemoglobin >130 g/L at the 2nd trimester of gestation [4], has been associated with prejudicial effects in the mother and fetus. These include preeclampsia and oxidative stress in the mother [9], as well as an increase in premature birth, low birth weight, and small-for-gestational-age [7,10,11] of the fetus.

Also, low birth weight and general poor neonatal health have also been associated with environmental factors such as tobacco consumption, toxic environment, low socio-economic status, and prenatal psychological status of the mother e.g. high levels of anxiety [12,13]. These factors, and others related to the process of iron supplementation and the effects on mother and child, need to be controlled in studies investigating iron levels and pregnancy.

Prophylactic iron supplements are prescribed to avoid iron deficiency during pregnancy. The health authorities of Spain recommend an iron supplementation of 30 mg/d for non-anemic pregnant women [14]. However, since this dose is not currently marketed, obstetricians recommend a dose of around 40 mg/d of iron.

However, there is still no consensus on the best dose, nor schedule of iron supplementation, to prevent its deficiency during pregnancy, without provoking hemoconcentration [1,4,15].

The lack of evidence on the best pattern of iron supplementation could correspond to other factors, apart from the dietary intake of iron, such as iron status of the woman during the first trimester [6,16-18], or the presence of a genetic alteration that modifies the intestinal absorption of iron.

With respect to iron status during the first trimester, when gestation begins with low levels of iron, the percentage of women with deficit at the end of pregnancy is much greater [6,16,17]. On the other hand, high levels of hemoglobin at the start of pregnancy in women taking high dose supplements (>60 mg/d) increases the risk of hemoconcentration by the end of gestation [4,10,19].

With respect to genetic alterations, the presence of the C282Y and H63D polymorphisms in the HFE gene have been associated with higher levels of hemoglobin [10,20]; a situation that can be aggravated if the individual is prescribed daily supplements of iron.

These factors (initial iron status and genetic alternations together with dietary intake and iron supplements) can explain the presence of different subpopulations of pregnant women, all having different iron needs during pregnancy. As a consequence, a prescribed iron dose modified to the individual’s characteristics would be needed to obtain optimum benefit.

There have been some studies that evaluated the effect of different doses of iron on iron deficiency as a function of the woman’s initial hemoglobin concentration but none, to the best of our knowledge, have also take into account possible iron excess and adverse effects on the newborn.

Hemoglobin is an ideal parameter to classify the women as a function of iron status, due to the high correlation with the levels of serum ferritin and with the presence of genetic alterations in the HFE gene. These measurements are relatively easy and quick, and are already used systematically in standard clinical practice, and in pregnancy follow-up.

Our hypotheses are that:

1) In women without anemia at the start of the pregnancy, with normal hemoglobin values of between 110–130 g/L, an iron intake of 40 or 80 mg/d, depending on the initial serum ferritin levels and on the presence of genetic alterations in the HFE gene, would be more effective in preventing iron deficiency, without increasing the risk of hemoconcentration by the end of pregnancy. This would help optimize mother-child health status.

2) In women without anemia and with normal-high values of hemoglobin of >130 g/L, an iron intake of 20 or 40 mg/d, depending on the initial serum ferritin levels and on the presence of genetic alterations in the HFE gene, will be more effective in ameliorating the risk of hemoconcentration by the end of the pregnancy, without leading to iron deficiency.

### Objective

The objective of the study is to determine the best grade of effectiveness (on mother-newborn health status), of an iron supplement adapted to the hemoglobin levels at the start of the pregnancy, relative to the usually-prescribed dose.

## Methods

### Trial design

Multi-centered, parallel groups, controlled, triple blind, randomized clinical trial (RCT) subdivided in 2 strata as a function of the hemoglobin (Hb) levels at the start of the pregnancy:

– Stratum #1: If the levels of Hb are situated between 110 and 130 g/L, the individual will be randomly assigned to an iron dose supplement of 40 or 80 mg/d

– Stratum #2: If the levels of Hb are >130 g/L, the individual will be randomly assigned to an iron dose supplement of 40 or 20 mg/d

**Figure 1:**
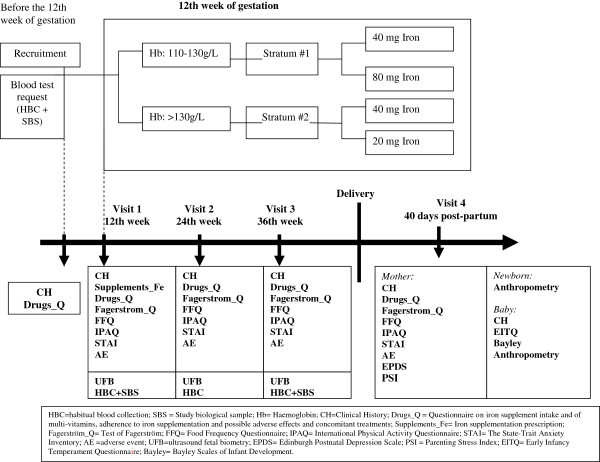
Study design.

The overall design of the study is summarized in Figure 1. This trial has been registered with EU Clinical Trials Register, EUCTR-2012-005480-28.

### Methods: participants, interventions, and outcomes

#### Study setting

The study will be conducted in 10 Primary Care Centers (PCC) of the Catalunya Sexual and Reproductive Health-care Service [*Atención a la Salud Sexual y Reproductiva (ASSIR)*] of the Catalan Institute of Health [*Instituto Catalán de la Salud (ICS)*]. These Centers provide care for 3,500 potential pregnancies per year. The specialist health-care workers include gynecologists and midwives. The participating reproductive health-care services (RHS) provide cover for urban, suburban, and rural PCCs.

### Eligibility criteria

The participants will need to fulfill the following inclusion, and none of the exclusion, criteria.

– Inclusion criteria: Adult female older than 18 years of age with ≤ 12 weeks gestation without anemia (Hb >110 g/L), capable of understanding the official State languages (Castilian or Catalan), who can understand the characteristics of the study, and who sign the informed consent form.

– Exclusion criteria: Multiple pregnancy, taking >10 mg iron during the months prior to week 12, hypersensitivity to egg protein (due to the iron prescription formula contains ovalbumin), previous serious illness (immunosuppressed status) or chronic illness that could affect the nutritional development (e.g. cancer, diabetes), malabsorption, and liver disease such as chronic hepatitis or cirrhosis. The risk factors of pregnancy to be considered as exclusion criteria are: adverse obstetric history, previous uterine surgery, heart disease grade 2, 3 or 4, endocrinopathy, suspected fetal malformation, morbid obesity, preeclampsia, maternal infection, uterine malformation, perinatal recurrent death, alcoholism, diabetes 1 and 2, uterine cervical incompetence.

### Interventions

The clinical follow-up of the pregnancy in the PCC will be according to the program set by RHS. This includes a clinical visit at recruitment into the present study, a visit every trimester, and one at 40 days post-partum (Figure 1).

In the recruitment visit before week 12 of the pregnancy, the inclusion criteria will be assessed (except the Hb levels and the number of fetuses) as well as the exclusion criteria. Informed consent will be solicited. A clinical history will be recorded, as will a questionnaire regarding the ingestion of iron supplements, multi-vitamins, or other treatments (Drugs_Q) and, if a smoker, the Fagerstrom test for tobacco dependency. A blood sample for standard biochemical analyses (including hemoglobin) will be sent for processing in the centralized laboratory.

At visit 1, around the 12th week of gestation, Hb levels will be evaluated as will be the number of fetuses (using echography) to confirm that the inclusion criteria are fulfilled. If fulfilled, the individuals will be retained in the study and, if not, will be transferred out of the study, and considered a screening failure. The remaining women will be assigned to Stratum #1 or Stratum #2 of the study and will be randomized with respect to iron supplement prescription. Clinical history will be taken, including the use of multi-vitamins and iron supplements (on the questionnaire abbreviated as “Drugs_Q”) and the following questionnaires filled-in: Food Frequency Questionnaire (FFQ); International Physical Activity Questionnaire (IPAQ); State-Trait of Anxiety Inventory (STAI) and the Fagerstrom questionnaire (Fagerstrom_Q). The ultrasound data on the fetus will be recorded. Venous blood will be taken for analyses, the results of which will be reviewed in the next clinical visit. The iron supplementation that will be needed at the next visit will be prepared for distribution. Adverse events occurring since the previous visit will be recorded.

At visit 2, around week 24 of gestation, clinical history will be taken, and will include the Drugs_Q, which, from this visit onwards, includes the adherence to the iron supplementation prescribed. The following questionnaires are filled-in: FFQ; IPAQ; STAI, Fagerstrom_Q. The fetal ultrasound data will be registered. The biochemical analyses/results will be reviewed and a further blood sample taken for analysis, the results of which will be reviewed at the next clinical visit. The iron supplementation that will be needed at the next visit will be prepared for distribution. Adverse events occurring since the previous visit will be recorded.

At visit 3, around week 36 of gestation, the clinical history will be taken, the Drugs_Q as well as the FFQ; IPAQ; STAI, Fagerstrom_Q questionnaires will be filled-in. The fetal ultrasound data will be recorded and the biochemical results will be evaluated. A further blood sample will be taken for analyses, the results of which will be discussed at the next clinical visit.

The iron supplementation that will be needed at the next visit will be prepared for distribution. Adverse events occurring since the previous visit will be recorded.

At visit 4 (40 days post-partum), the clinical history will be taken, the Drugs_Q and the FFQ; IPAQ; STAI; Fagerstrom_Q questionnaires will be filled-in. A questionnaire on post-partum depression (Edinburgh Postnatal Depression Scale; EPDS) and the Parenting Stress Index (PSI) will be applied. The standard laboratory analyses results will be discussed. A further blood sample will be taken for analyses. Data on birth and the newborn will be recorded (weight and height). Clinical history of the baby will be recorded, including: anthropometric data (weight, height, head circumference) and cognitive development will be assessed (Bayley III), as well as behavioral and temperament (Early Infancy Temperament Questionnaire; EITQ). Adverse events occurring since the previous visit will be recorded.

#### Iron supplementation

Starting from around the 12th week of gestation and continuing up to partum, adherence to prescription will be recorded. The dose assignments will be random and followed-up blind for each RCT stratum. The principal active ingredient is ferrimanitol ovoalbumin. The doses of 20 mg, 40 mg and 80 mg per day of elemental iron correspond to 150 mg, 300 mg and 600 mg ferrimanitol ovoalbumin. The reference dose is 40 mg/d, similar to that routinely prescribed in clinical practice [14].

#### Biological samples of the RCT

The blood extractions at visits 1, 3 and at birth, will be more than are normally taken for biological analyses in a RCT. This is because slightly larger amounts are required for subsequent analyses such as specialized biochemical measurements (ferritin, cortisol) and genetics (C282Y and H63D of the HFE gene). These samples will be treated and stored for subsequent analyses in the BioBank of the reference hospital of the participating PCCs.

#### Participant withdrawal

Participants will be free to withdraw from the trial at any time, and without the need to provide any reason. The information that may already have been collected on participants will still be used. Withdrawal from the study will not affect the standard of care received from the practice. If participants withdraw before randomization, they will be replaced by an appropriate substitute.

The pregnancies diagnosed as anemic during the RCT will have the iron supplementation from the RCT changed, and will be treated according to the standard clinical practice protocol. These women will continue forming part of the study and will have the same follow-up, except they will be considered as therapeutic failures in the intent-to-treat statistical analyses, and will not be taken into account in the protocol analysis.

#### Discontinuation visit

This visit will take place only in case of the participant’s withdrawal from the study at any time outside the scheduled timetable. The exact date and reason for discontinuation will be registered. If possible, the healthcare professional will assess the woman’s health status via clinical history notes, and an ultrasound. A blood sample will be solicited for routine tests. The adherence to the iron supplementation up until the discontinuation of the treatment will be recorded, as will any adverse events. All the questionnaires will be filled-in, including: FFQ; IPAQ; STAI, Fagerstrom_Q. In case of having given birth, all the variables related to delivery and the newborn will be collected and a questionnaire on post-partum depression (Edinburgh Postnatal Depression Scale; EPDS) and the Parenting Stress Index (PSI) will be applied.

### Outcomes

#### Primary outcomes

Principal variables in the pregnancy are iron status of the mother at each clinical visit (anemia, ferropenic anemia, risk of hemoconcentration) and birth weight of the baby.

Definition of main outcome variables

– Anemia is defined as Hb <110 g/L in the 1st and 3rd trimester, Hb <105 in 2nd trimester, Hb <120 g/L post-partum [21].

– Ferropenic anemia is defined as: Hb < the normal limit, and serum ferritin (SF) <15 μg/L [22].

– Hemoconcentration risk is defined as: Hb >130 g/L in the 2nd and 3rd trimester [4].

### Participant timeline

Clinical follow-up of the pregnancy in the PCC will be according to the program set by ASSIR. The participant timeline is detailed in Figure 1

– Recruitment visit (previous to week 12 of gestation)

– 1st visit of the study (around 12th gestational week)

– 2nd visit of the study (around 24th gestational week)

– 3rd visit of the study (around 36th gestational week)

– 4th visit of the study (40 days post-partum)

### Sample size

The principal variables taken into account in calculating the size of sample needed are iron deficiency anemia, risk of hemoconcentration in the third trimester of pregnancy, and birth weight of the baby.

To achieve the study’s main objective, sample size is calculated in accordance with the following parameters: an alpha risk of 0.05 and a beta risk of 0.20 in a one-tailed test of comparison. A drop-out rate of 35% is factored-in.

To calculate the sample size needed to reduce the prevalence of iron deficiency anemia and hemocentration risk by 10 points, we consulted previous data from our research group [5,6,8]. We had observed a prevalence of 23.5% of iron deficiency anemia in the 3rd trimester in pregnant women with Hb levels of 110-130 g/L in the first trimester and a prevalence of risk of hemoconcentration of 14.7% in the 3rd trimester of pregnant women who started pregnancy with Hb levels of 130–150 g/L.

We found that, in Stratum #1 of the RCT, it will be necessary to include 283 women in each group and in Stratum #2 of the RCT 156 women would be necessary in each group.

With respect to child development, to detect differences in weight of the newborn ≥200 g taking into account a standard deviation (SD) of 450 g [5], the estimation suggests that it will be necessary to have 106 women in each group.

Hence, the overall study sample is calculated as 878 women: 566 in Stratum #1 and 312 in Stratum #2.

### Methods: intervention assignment

#### Allocation

The pregnant women are assigned to Stratum #1 or Stratum #2 as a function of the hemoglobin values in the baseline analysis of the study. They are, then, randomly assigned to 2 treatment groups to receive different iron supplements. The randomization is performed using centralized computer software, which is automatic and masked and applies to the electronic data collection forms, as well. The procedure for randomization is independent for each Stratum.

### Blinding

The study will be triple blind: the participant, the health-care professional, and the statistician. The treatment drug will be administered “blind” i.e. the doses are not identifiable since the packaging has the same format, presentation, and visual characteristics.

For Stratum #1, the treatments will be designated as A or B, and for Stratum #2 they will be designated as C or D.

The laboratory of MEIJI TEDEC FARMA, SA will be responsible for manufacturing, packaging and labeling the study medications.

Only MEIJI TEDEC FARMA, SA and the Clinical Pharmacology Service of the Vall d’Hebron Hospital in Barcelona will know the distribution codes and the composition of each of the treatments.

There would be no need for un-blinding except if an unexpected serious adverse event occurs. In which case, the pharmaco-vigilance staff of TEDEC-MEIJI FARMA S.A. will take responsibility for un-blinding and communicating the adverse event to the appropriate health authorities. TEDEC-Meiji Farma SA will not reveal the treatment codes until the end of the trial, when these data and the documents generated will be made available to the Principal Investigator (VA) and the Promoter (Jordi Gol i Gurina).

### Methods: data collection, management and analyses

#### Data collection methods

Midwifes will be responsible for data collection from the clinical history, and from the questionnaires. They will introduce the data into an electronic data collection format which will be monitored by an external service.

The ultrasound measurements will be conducted by two obstetricians using previously-standardized techniques.

The pregnancy

– Clinical history of the mother: date of birth, socio-economic status, parity, date of last menstruation, corrected date of last menstruation, estimated date of partum, risk factors during pregnancy, pregnancy planning, previous use of contraceptives, clinical antecedents, surgery and personal obstetric data, toxic habits, blood pressure, height, weight of the mother (self-reported at the recruitment visit and measured objectively at each clinical follow-up visit). Similar data from the father will be solicited.

– Questionnaire on iron supplement intake and of multi-vitamins, adherence to iron supplementation and possible adverse effects and concomitant treatments (Drugs_Q)

– Food Frequency Questionnaire (FFQ) validated by our research team [23]

– International Physical Activity Questionnaire (IPAQ) [24,25]

– State-Trait Anxiety Inventory (STAI) [26]

– Fagerstrom test to evaluate tobacco dependency (Fagerstrom_Q) [27]

– Edinburgh Postnatal Depression Scale (EPDS) [28,29]

– Parenting Stress Index (PSI) [30,31]

– Standard laboratory analyses: Hemogram: hemoglobin, mean corpuscular volume, hematocrit (Coulter, Hialeah, FL, USA)

– Biological samples for the measurement of biochemical parameters (ferritin, cortisol) and genetic polymorphisms (C282Y and H63D of the HFE gene)

#### The fetus

Ultrasound data

– Ultrasound #1: confirmation of the date of the last menstruation and date of gestation corrected for echography, prenatal diagnostics, chorion characteristics, amniotic fluid and determination of secondary markers

– Ultrasound #2: biometry, morphological evaluation, gender, position and presentation of the fetus, body and cardiac activity, characteristics of the placenta and umbilical cord, and status of the fetus

– Ultrasound #3: evaluation of fetal growth using biometry, body and cardiac activity, characteristics of the placenta, of the umbilical cord, and position and status of the fetus

The newborn

– Clinical history of the newborn (gender, status of the newborn, weight and height at birth) and anthropometric measurements of weight, height, head circumference at 40 days

– Bayley III scales: indices to evaluate mental and psychomotor development [32]

– Early Infancy Temperament Questionnaire (EITQ) [33]: test to evaluate behavior and temperament

#### Procedures for extraction, transfer and storage of biological samples

Blood samples will be collected in 2 tubes; 1 tube of 7.5 ml containing EDTA as anticoagulant and 1 tube of 7.5 ml without anticoagulant. The samples will be transported to the BioBank for immediate analyses to generate the hematology profile using the laboratory’s Coulter analyzer. In the BioBank, the samples will be prepared for storage. Before processing, the samples in the EDTA tubes will be inversion-mixed 10 times to ensure that the blood is well mixed before centrifugation at 4°C to separate plasma which will be stored in aliquots (500 μl) at -80°C for measurements described later. DNA will be extracted and stored at -80°C for subsequent genetic analyses. The tube without anticoagulant will be left without mixing for 30 minutes at room temperature to enable coagulation. The serum will be separated by centrifugation, distributed in aliquots of 500 μl and stored at -80°C.

The stored samples in the BioBank will be thawed at the end of the clinical study and processed simultaneously to minimize inter-batch variation:

1) Serum ferritin (immunochemiluminescence)

2) C-reactive protein (immunoturbidimetry)

3) Cortisol (immunochemiluminescence)

4) Genetic analyses (only in the first blood sample): mutation C282Y, H63D of the HFE gene (PCR and digestion with specific enzymes)

To control for possible bias, the intra- and inter-assay coefficients of variation will be calculated for all the measured variables. Values recommended by the “kit” manufacturers are accepted. All the biochemical determinations of all the samples in a series will be performed by the same person so as to minimize inter-assay variations in the longitudinal measurements of variables that are of interest to the investigators.

### Statistical methods

The description of the variables studied will be performed using conventional techniques. Variables with non-normal distribution will be transformed as necessary for normalization of distribution of values. The Kolmogorov-Smirnov and the Shapiro-Wilks test will be used to verify normality of distributions.

#### Analysis of the primary outcome

The effects of iron dose supplement in each RCT on the biochemical iron status and mother-child health will be compared using regression models adjusted for those variables that can influence the relationship. Logistic regression or Cox models will be applied for qualitative variables such as, for example, the percentage of anemia or hemoconcentration at the end of pregnancy. Linear multiple regression models will be applied for dependent quantitative variables. The models will be adjusted for those variables that biologically affect the relationships studied, such as the serum ferritin levels, presence of alterations in the HFE gene, age of the mother, gestational age, parity, anthropometric indices, diet, and lifestyle, and the interactions between these variables. Initially included in the model will be all those variables that form part of the theoretical model and, in a second phase, the variables for entry into the model will be selected step by step (forward and backward) to achieve the most reduced stable models.

Conditions for the application of models will be verified using standard techniques that are based, essentially, on residuals analysis. The bilateral null hypothesis of normality, no difference, and non-significance of the regression coefficients, will be rejected when their probabilities are <5%. The data will be analyzed using the latest version of SPSS/PC package for Windows.

Data will be analyzed in 2 ways:

a) Intention-to-treat analysis

The principle of “intention-to-treat” is a way of analyzing the results that considers all individuals entered into the study, according to the group to which they were originally assigned, although they may not have complied with the protocol. In this case, in all the circumstances that have led to not having hemoglobin determinations before delivery, we will proceed to incorporate at this time-point the immediately-prior determination of hemoglobin. All randomized participants randomized who had received at least one dose of study medication will be included.

b) Per-protocol analysis

Included in the analysis will be only those pregnant women who received the allocated intervention at randomization. The per-protocol population (PP) will include those participants who had taken at least the 80% of the study medication, with proper assessment of adherence, or without any major protocol violations.

Pregnant women diagnosed with anemia at the start will be treated according to the usual protocol, and not as a part of the current study. Women diagnosed with anemia posteriorly, will be treated with standard therapy, but will remain part of the study in the intention-to-treat analysis. However, they will not be considered in the per-protocol analysis.

Data missing from the study variables will be treated with the direct likelihood method [34] when no data is available. In these cases, the profile of pregnant women will be used to adjust the estimates of these parameters.

An analysis of sensitivity will be performed using a mixed model based on constraint-based patterns of missing values without dependence on the future to assess the robustness of the main results of the Mixed Model Repeated Measures against the possible transgression of random default assumption.

### Monitoring

To ensure correct conduct and security of the RCT according to the requirements of good clinical practice, external services will be contracted to perform the tasks of monitoring of the participating centers according to the requirements of the Spanish Agency of Medicines and Health Products [*Agencia Española de Medicamentos y Productos Sanitarios; AEMPS*].

### Harms

All adverse events suffered will be recorded at each clinical visit and assessed for severity, expectedness and causality.

### Ethics and dissemination

#### Research ethics approval

In Spain, approval was obtained from the Clinical Research Ethics Committee of the Jordi Gol Research Institute in Primary Care *[Instituto de Investigación en Atención Primaria; IDIAP]* and from the Spanish Agency for Medicines and Health Products *[Agencia Española del Medicamento y Productos Sanitarios; AEMPS]*.

The study is designed according to the requirements of the Declarations of Helsinki, and the Tokyo update.

### Consent

All participants are informed of the study, its objectives and activities related to their participation: number and schedule of visits, diagnostic tests, results information. Patients will be provided with detailed information leaflets, and will be asked to sign a consent form.

### Confidentiality

Confidentially will be guaranteed for all participants in the study. The study conforms to the Government Data Protection Act for Personal Information (15/1999 of 13th Dec. LOPD).

### Access to data

All documents will be stored securely and accessible only to trial staff and authorized personnel.

### Dissemination policy

All scientific publications must have the approval of the principal investigator (VA).

The principal investigator of the study is committed to making public such results as become available, both positive and negative. When studies and research are made public to the scientific community, the funding sources and the laboratory TEDEC-MEIJI FARMA S.A will be acknowledged.

The researchers are committed to making raw, anonymity-guaranteed, data sets available to the scientific community upon legitimate request to the principal investigator, once the trial is completed.

## Discussion

The ECLIPSES randomized clinical trial is an intervention study in Primary Care directed towards pregnant women. It attempts to establish the optimum iron supplementation to prevent not only iron deficit but also iron excess during gestation. The approach used is to adapt the standard supplementation to the individual characteristics of the pregnant woman, based on the hemoglobin level at the start of gestation.

Prevention is important, given the elevated percentage of anemia during pregnancy (around 25%) and of the risk of hemoconcentration (around 15%); conditions which have been associated with adverse effects not only in the mother but also in the newborn.

We will take the following measures to minimize, or avoid, bias in the conduct of the trial:

– Randomization: An externalized randomization program will be used to ensure allocation concealment

– Use of an electronic data collection system that enables homogeneous data collection and reduces transcription errors

– External RCT monitoring, which would increase the reliability of the results

– Blinding and assessment of outcomes: Iron supplements administration would be blinded. Therefore, neither the investigators nor the participants in the study nor the statistician would know the dose of iron in each of the treatment groups.

– Combined batched analyses of samples at the end of the study. The samples will be stored at -80°C in the Hospital’s BioBank and analyzed together at the end of the study, thereby limiting the potential bias that could be introduced by several different analytical procedures and laboratory personnel turn-over in the course of the trial. Further, inter- and intra-assay coefficient of analysis will be calculated for all of the measurements performed.

– Standardization of the activities of the health-care professionals involved in the different aspects of the study. Before starting the study, all the health-care professionals participating in the study will standardize their procedures so as to ensure that all the tests and/or questionnaires are conducted in the same manner.

The particular challenges that we anticipate in this study are as follows:

– Loss to follow-up. We hope to reduce this by taking advantage of the clinical visits that the pregnant women would normally have with her gynecologist/obstetrician. As such, she would not be required to undertake additional activities/visits by participating in the current trial. Further, we have increased the study sample size so as to compensate for possible loss to follow-up.

– The supplementation with iron can produce gastrointestinal side effects that can affect the adherence to treatment. To reduce this effect, the principal component of the supplement will be ferrimanitol ovalbumin which has been shown to have greater bioavailability and better tolerance than ferrous sulfate.

– It is difficult to know the adherence to the prophylactic iron supplement. To improve the registry, we will apply a semi-structured questionnaire on the degree of adherence to the prescription in all the clinical visits during pregnancy. This will indicate the number of units that have been returned to the investigator each week.

A primary strength of the study is its rigorous methodology that involves a large number of health-care professionals and patients, which will strengthen the validity of the findings. Conclusive outcomes would help the obstetrician in deciding the best iron supplementation pattern required to promote maternal and infant health. Initial hemoglobin levels that are routinely collected on all pregnant women attending the clinic, and there will not be any additional procedures that could dissuade participation by the pregnant woman.

Our findings would contribute to developing guidelines for good clinical practice.

## Abbreviations

Drugs_Q: Multi-vitamins and iron supplements questionnaire; EITQ: Early infancy temperament questionnaire; EPDS: Edinburg postnatal depression scale; Fagerstrom_Q: Fagerstrom questionnaire; FFQ: Food frequency questionnaire; Hb: Hemoglobin; ICS: Catalan institute of health; IPAQ: International physical activity questionnaire; PCC: Primary care centers; PSI: Parenting stress index; RCT: Randomized clinical trial; RHS: Reproductive health-care services; STAI: State-trait of anxiety inventory.

## Competing interests

The authors declare that they have no competing interests.

## Authors’ contributions

VA, FF, GM, SA, JB, JC, BR, EA, NS, CH and NA are all members of the ECLIPSES Study Group. VA, FF and NA conceived the study and participated in the project design. VA: as principal investigator for the ECLIPES study, additionally wrote the first draft of the ECLIPES protocol and prepared the initial draft of the current manuscript. All authors were involved in the design and development of the study, writing the protocol, and critically reviewing all drafts of the protocol. All authors have read and approved the final version of the protocol/manuscript.

## Pre-publication history

The pre-publication history for this paper can be accessed here:

http://www.biomedcentral.com/1471-2393/14/33/prepub
